# CIRCADIAN CLOCK DISRUPTION: SEX-SPECIFIC EFFECTS ON METABOLISM & COGNITION IN AN ALZHEIMER’S MODEL

**DOI:** 10.1093/geroni/igad104.3225

**Published:** 2023-12-21

**Authors:** Laila Scroggins, Qing Zhang, Kevin Koronowski, Juan Palavicini

**Affiliations:** Howard University, Washington, District of Columbia, United States; ​The Barshop Institute for Longevity and Aging Studies, San Antonio, Texas, United States; ​The Barshop Institute for Longevity and Aging Studies, San Antonio, Texas, United States; ​The Barshop Institute for Longevity and Aging Studies, San Antonio, Texas, United States

## Abstract

Preclinical studies have been largely performed on transgenic murine models of early-onset AD, which only accounts for a small fraction of AD cases. A number of confounds have impacted and limited the translatability of these mouse models, including potential off-target genetic effects, artifacts resulting from ultra-physiological overexpression of specific gene products, and overblown pathologies in non-fully developed brains. The objective of this study is to leverage a new microtubule-associated protein tau knockin (MAPT H1-GR) mouse model to assess the effects of an understudied environmental factor associated with AD, a disturbed circadian rhythm, on whole body and brain metabolism, tauopathy, neuroinflammation, neurodegeneration, and cognition. For the experimental design, a cohort of 32 mice was divided into two groups, normal light conditions (NLC) and chronic jet lag (CJL). The CJL mice have a regulated light schedule which shifts 4 hours ahead every 3 days while the NLC mice have a continuous 12 hours of light and 12 hours of dark schedule. Non-fasted and overnight-fasted glucose, ketones, and lactose levels were measured. CJL led to a sex-dependent disruption of fuel utilization during fasting conditions with males displaying more dramatic phenotypes than females. CJL also led to sex-specific changes in body composition, assessed via qMRI testing every other month, specifically at the level of fat mass in males. Cognitive assessment through the Barnes Maze test revealed a disruption of spatial memory in males, while females seemed to be protected from the deleterious effects of CJL on cognition.

